# Management of recurrent vulvovaginal candidosis: Narrative review of the literature and European expert panel opinion

**DOI:** 10.3389/fcimb.2022.934353

**Published:** 2022-09-09

**Authors:** Gilbert Donders, István Oszkár Sziller, Jorma Paavonen, Phillip Hay, Francesco de Seta, Jean Marc Bohbot, Jan Kotarski, Jordi Antoni Vives, Bela Szabo, Ramona Cepuliené, Werner Mendling

**Affiliations:** ^1^ Femicare VZW, Clinical Research for Women, Tienen, Belgium; ^2^ Department of Obstetrics and Gynecology, University Hospital Antwerp, Antwerp, Belgium; ^3^ Department of Obstetrics and Gynecology, Regional Hospital Tienen, Tienen, Belgium; ^4^ Dél-budai Centrumkórház, Szent Imre Egyetemi Oktatókórház, Szülészet és Nőgyógyászati Osztály, Budapest, Hungary; ^5^ Department of Obstetrics and Gynecology, Helsinki University Hospital, Helsinki, Finland; ^6^ Guys and St. Thomas’ NHS Foundation Trust, London, United Kingdom; ^7^ Department of Medical, Surgical and Health Sciences, Institute for Maternal and Child Health, University of Trieste, IRCCS Burlo Garofolo, Trieste, Italy; ^8^ Department of Sexually Transmitted Infections, Institut Alfred Fournier, Paris, France; ^9^ Department of Oncological Gynecology and Gynecology, Medical University of Lublin, Lublin, Poland; ^10^ Department of Gynecology and Obstetrics, Hospital CIMA, Barcelona, Spain; ^11^ Department of Obstetrics-Gynecology, “George Emil Palade” University of Medicine, Pharmacy, Science and Technology, Targu-Mures, Romania; ^12^ Gedeon Richter Plc., Budapest, Hungary; ^13^ Deutsches Zentrum für Infektionen in Gynäkologie und Geburtshilfe, Helios Universitätsklinikum Wuppertal, Wuppertal, Germany

**Keywords:** recurrent vulvovaginal candidosis, maintenance regimen, azole therapy, antifungal, routine clinical practice, expert opinion

## Abstract

Recurrent vulvovaginal candidosis (RVVC) is a chronic, difficult to treat vaginal infection, caused by *Candida* species, which affects women of all ages and ethnic and social background. A long-term prophylactic maintenance regimen with antifungals is often necessary. In most clinical practice guidelines, oral fluconazole is recommended as the first-line treatment. Although clinical resistance to antifungal agents remains rare, overexposure to azoles may increase the development of fluconazole-resistant *C*. *albicans* strains. In addition, *non-albicans Candida species* are frequently dose-dependent susceptible or resistant to fluconazole and other azoles, and their prevalence is rising. Available therapeutic options to treat such fluconazole-resistant *C. albicans* and low susceptibility non-*albicans* strains are limited. Ten experts from different European countries discussed problematic issues of current RVVC diagnosis and treatment in two audiotaped online sessions and two electronic follow-up rounds. A total of 340 statements were transcribed, summarized, and compared with published evidence. The profile of patients with RVVC, their care pathways, current therapeutic needs, and potential value of novel drugs were addressed. Correct diagnosis, right treatment choice, and patient education to obtain adherence to therapy regimens are crucial for successful RVVC treatment. As therapeutic options are limited, innovative strategies are required. Well- tolerated and effective new drugs with an optimized mechanism of action are desirable and are discussed. Research into the impact of RVVC and treatments on health-related quality of life and sex life is also needed.

## Introduction

Recurrent vulvovaginal candidosis (RVVC) is a debilitating, chronic vaginal infection (Denning et al., 2018) caused by *Candida albicans* (*C. albicans*) and non-*albicans* pathogenic fungus species (Sherrard et al., 2018; Farr et al., 2021a). It is an incurable condition affecting healthy women of all ages and social strata (Lema, 2017; Willems et al., 2020; Farr et al., 2021a).

RVVC is inconsistently and arbitrarily defined in the clinical practice guidelines (Denning et al., 2018). For instance, in the United States (US), RVVC is defined as three or more episodes of symptomatic vulvovaginal candidosis (VVC) in less than one year (Workowski et al., 2021). In the European guidelines and according to the Infectious Diseases Society of America guidelines, four or more symptomatic episodes of VVC per year are needed for this diagnosis (Pappas et al., 2015; Sherrard et al., 2018; Farr et al., 2021a; McKloud et al., 2021).

It is generally accepted that two forms of RVVC exist (Lema, 2017). Primary RVVC is idiopathic, with unknown predisposing factors, and occurs in otherwise healthy immunocompetent women of whom the majority has no discernible precipitating or causative factors. Secondary RVVC, in contrast, has known predisposing factors including an estrogenized vagina (postmenarchal and premenopausal), postmenopausal hormone replacement therapy, diabetes mellitus, atopy, antibiotic/corticosteroid use, or pregnancy (Fischer and Bradford, 2011; Lema, 2017). Infection with the human immunodeficiency virus (HIV) is also associated with more frequent episodes of RVVC (Fischer and Bradford, 2011; Alczuk Sde et al., 2015; Mohammed et al., 2021).

The burden of RVVC is underestimated. It compromises women’s quality of life and is associated with high morbidity and medical care costs (Lema, 2017; Rosati et al., 2020; Willems et al., 2020), reduced physical and psychological wellbeing, and impaired sexual activity and satisfaction (Mohammed et al., 2021). The employment and financial resources of women with RVVC are also threatened (Fukazawa et al., 2019).

The management objectives for patients with RVVC include elimination of potentially reversible risk factors (Donders et al., 2011), providing rapid symptomatic relief, clearance of the pathogen from the female genitalia, and prevention of repeated episodes (Lema, 2017). Topical or oral antifungal azole-based formulations are widely recommended (Pappas et al., 2015; Sherrard et al., 2018; Farr et al., 2021a). Compared with placebo or no therapy, treatment with oral or topical antifungals reduces symptomatic clinical recurrences (Cooke et al., 2022). Although episodic fluconazole treatment is at times sufficient to prevent frequent recurrences, long-term antifungal prophylaxis with a maintenance regimen is often necessary (Sobel and Sobel, 2018). However, differences between treatment options (e.g., different doses and durations) that may explain variations in their efficacy or effectiveness remain imprecisely defined (Cooke et al., 2022).

Although clinical *C.* *albicans* resistance, either inherent or acquired, toward antifungal agents continues rare, overexposure to treatment in such maintenance regimens and regular use of over the counter (OTC) or prescribed therapies may increase the potential for the development of fluconazole-resistant strains, as demonstrated in *in vitro* minimal inhibiting concentration passaging studies (Mathema et al., 2001; Blostein et al., 2017).

In line with this, epidemiological data confirm that almost all women diagnosed with resistant *C.* *albicans* strains had previously been exposed to fluconazole (Marchaim et al., 2012). In contrast to *C.* *albicans*, non-*albicans Candida* species frequently show reduced susceptibility, meaning that higher doses are required to achieve antifungal effects or are resistant to fluconazole and other azoles (Sobel and Sobel, 2018; Ignjatovic et al., 2020). Currently, available therapeutic options to treat fluconazole-resistant *Candida *species are limited (Sobel and Sobel, 2018; Farr et al., 2021a; Van Riel et al., 2021). RVVC is therefore a distressing condition for patients and a challenging long-term disease for healthcare professionals (Sustr et al., 2020).

The management of RVVC was discussed among 10 international European experts in gynecology who are the authors of this paper. Discussions took place during two online meetings held between May 2021 and April 2022. In the present paper, the authors review the relevant literature and report on the outcomes of these meetings. The aim of the present overview is to define the clinical and mycological effectiveness of current management protocols for RVVC and to identify possible therapeutic pitfalls leading to suboptimal cure rates.

## Materials and methods

A group of 10 experts from Belgium, Finland, France, Germany, Hungary, Italy, Poland, Romania, Spain, and the UK participated in two online sessions and two follow-up rounds to identify current practice in RVVC diagnosis and treatment, to define the profile of patients with RVVC and their care pathways, to address current therapeutic needs, and to discuss the potential value of novel drugs coming onto the market. The overall aim was to understand the real-world management of RVVC from the perspective of experts in the field.

Participants were selected because of their national and international standing as opinion leaders and experts in gynecological infections, their regular participation as speakers and authors at scientific events and in publications, and their contributions to guidelines, working groups, and educational initiatives. All opinion leaders were invited to sign in an official internet-based platform (http://within3.com). Specific questions were discussed and moderated by the lead author (GD).

During the first session held in May 2021, a total of 33 open-ended questions were posed by three moderators. An online survey was run simultaneously in which experts responded to specific questions on the condition, such as the average age of women with RVVC, how an acute VVC episode is distinguished from a non-acute one, frequency of self-treatment, treatment options most used, reasons for treatment switching, factors contributing to the success of treatments, and compliance. Statements were rated according to their relevance or frequency in common practice.

The sessions were recorded and transcribed. A total of 340 statements were summarized. The opinions were streamlined and summarized as an expert-based opinion.

During the second session held in October 2021, experts were presented with a summary of the results of the first meeting and asked to comment until agreement was reached on their accuracy. A round of follow-up questions went along. Findings are presented below and compared with evidence from the literature.

## Results

### The disease

#### The epidemiology

RVVC affects approximately 138 million women per year worldwide (projected to reach an estimated 158 million by 2030), with a global annual prevalence of 3,871 per 100,000 women (Denning et al., 2018). The highest prevalence rate (9%) is observed in women aged 25 to 34 years, with a global prevalence of about 7% in 15- to 54-year-old women (Denning et al., 2018; Sherrard et al., 2018; Farr et al., 2021a). Results from an internet survey among ~6,000 women from France, Germany, Italy, Spain, the UK, and the US revealed a similar population-based RVVC prevalence of 9% (Foxman et al., 2013).

Numbers are highest in the most populous countries. The number of cases per 100,000 women in the Americas (4,060 per 100,000) and Asia (4,040 per 100,000) is higher than in Europe with an average of 3,755 per 100,000. Infection rates range from in 3,543 in France to 3,961 per 100,000 women in Spain (Denning et al., 2018). According to a recent study, the probability of women developing RVVC in European countries before the age of 50 is 24% (Blostein et al., 2017).

*C. glabrata* has more recently been referred to as *Nakaseomyces glabrata* and *C.* *krusei* as *Pichia kudriavzevii* in the revised nomenclature of fungi of medical importance (Borman and Johnson, 2021). Despite their traditional names, these two species do not belong to *Candida* spp. and are therefore less pathogenic for healthy, immunocompetent women (Gabaldón and Fairhead, 2019). *C. glabrata*, genetically a Saccharomyces relative, is generally overestimated as a vaginal pathogen, as it is nearly always a colonizer especially in peri- and postmenopausal women (Mendling et al., 2015). As the new nomenclature is not yet accepted worldwide and, currently, would cause confusion, the panel decided to use the traditional names in the current discussion. In a retrospective polymerase chain reaction (PCR)–assisted analysis of 93,775 cervicovaginal smears, of all positive samples, the prevalence of *C. albicans* was 89%, whereas *C. glabrata* represented 7.9% of cases (Vermitsky et al., 2008). *C.* *albicans* accounts for the majority of all documented RVVC cases (74%), followed by the non-*albicans* species, including *C.* *glabrata* and *C.* *krusei* found in about 14% and 6% of RVVC cases, respectively (Ignjatovic et al., 2020).

Recently, the prevalence of RVVC has increased, partly due to a rise in VVC caused by non-*albicans* species (Makanjuola et al., 2018; Ignjatovic et al., 2020). Non-*albicans* species can be present in a significant number of cases in women of reproductive age, according to recently published literature (Narayankhedkar et al., 2015; Moreira et al., 2021). The distribution of species among different age groups reveals an increase in the percentage of non-*albicans* species with increasing age (Vermitsky et al., 2008). Fungal infections of the lower genital tract dramatically decrease during menopause, except for women using hormonal replacement therapy and for post-menopausal women with poorly controlled diabetes irrespective of their hormonal status (Nwokolo and Boag, 2000). These women typically have higher rates of colonization with non-*albicans* species (Kim and Park, 2017; Lema, 2017; Makanjuola et al., 2018).

*In the experience of the meeting experts, fertile women are the most frequently affected with the highest frequency in those between 25 and 40 years of age. In line with reports from the literature* (Hoffmann et al., 2014; Al Halteet et al., 2020)*, experts also found that menopausal women only rarely develop Candida infections and that this mostly occurs after using antibiotics, with poorly controlled diabetes, or when on hormone replacement therapy.*

### The host

RVVC is considered a multifactorial disorder, the symptoms of which are governed by the interaction between *Candida* (species and virulence factors), the Lactobacilli population, the microenvironment (estrogen, inflammatory status, and oxidative stress), and the host (genetic factors, immune status, and behavioral factors). A disrupted balance in these factors may increase the susceptibility to RVVC (Rosati et al., 2020; Van Riel et al., 2021).

Pregnancy, contraceptives, diabetes mellitus, antibiotic use, use of poorly ventilated clothing, frequency of sexual intercourse, casual sex, regular oral sex, bacterial vaginosis, atopic disease, immunosuppressive regimens, HIV infection, vulvar dermatosis, and genetic predisposition are factors that promote vaginal infection in some women, but not in all (Hellberg et al., 1995; Sovianti and Devi, 2021).

Single-nucleotide polymorphisms and other genetic alterations effecting key signaling proteins in the host may prompt the onset of VVC and enhance susceptibility to RVVC (Rosati et al., 2020; Bojang et al., 2021). Genetic variants in pattern recognition receptors or in signal transducers have been found to impair the antifungal immune response in patients with RVVC (Rosati et al., 2020). Phagocytes play a key role in clearing *C.* *albicans*, which is primarily mediated by pathogen-associated molecular pattern—pattern recognition receptor interactions to trigger the activation of innate immune cells (Yano et al., 2018). Symptomatic vaginal infections are characterized by a nonprotective proinflammatory innate immune response, mediated by the influx of neutrophils (Willems et al., 2020; Bojang et al., 2021). However, these recruited neutrophils are unable to phagocytize and clear the fungal pathogen, leading to an amplified nonprotective proinflammatory response (Bojang et al., 2021). The neutrophil dysfunction in VVC and RVVC has been linked to the inability of complement receptor 3 to interact with fungal cell wall proteins, promoting *Candida* survival and symptomatic infection in patients with VVC and RVVC (Soloviev et al., 2011; Bojang et al., 2021).

Vaginal estrogen levels play an important role in predisposing women to the disease. Fertile women in their child-bearing years, with high estrogenic phases during their reproductive cycle, are most susceptible to disease onset (Willems et al., 2020). Age, previous illness, a weakened or altered immune system, impaired tolerance to glucose, and severe immunosuppression are proposed risk factors for the development of recurrent disease (Donders et al., 2002; Donders et al., 2008b). Furthermore, women with a family history of atopy, prolonged symptom duration, and severe vaginal excoriation have an increased risk of not responding to fluconazole maintenance therapy (Donders et al., 2018a), whereas patients with colonization at multiple body sites, especially in the mouth and/or anus, also have a tendency to respond less well to maintenance therapy (Donders et al., 2018b). Predisposing factors are absent in 20%–30% of patients with RVVC (Rosati et al., 2020). It is suggested that the *Candida* strain, its virulence, and inter-individual differences determined by ethnicity, genetic mutations, and polymorphisms are crucial in the pathogenesis of this form of idiopathic RVVC (Rosati et al., 2020).

### The communication between the host and fungal organisms

The communication between the host and the fungal organism is complex, leading to overreaction and hypersensitive responses in some women and to a downregulation of symptoms in others. Most of these reactions are mediated through inflammatory pathways. One of these pathways is the activation of the NLRP3 (NLR family Pyrin protein 3) inflammasome, leading to unconstrained production of interleukin-1β (IL-1β) and recruitment of neutrophils. In mouse models, IL-9 not only exerts inflammation by promoting this NLRP3 inflammasome during the initial stages of VVC but also increases tolerance against infection toward the recovery phase by inducing the IL-1 receptor antagonist (Renga et al., 2018). To dampen this reaction, IL-18 and IL-22 are required *via* the NLRC4 inflammasome/IL-1Ra axis. In the IL-18–deficient mice, the sensitivity to VVC is increased. On the other hand, stimulating IL-22 was found to decrease IL-18 and to offer protection against VVC (Borghi et al., 2019).

In trying to unravel the mechanism of the transition from colonization to symptomatic disease, Roselletti and collaborators discovered that the level of Toll-like receptor 4 (TLR4), TLR2, and erythropoietin-producing hepatoma A2 (EphA2) expression was significantly higher in epithelial cells from *Candida* infected subjects than from healthy subjects, whereas activation of nuclear factor–κB (NF-κB) and c-Fos-p-38 occurred in epithelial cells from symptomatic pseudohyphae/hyphae carriers but not in asymptomatic yeast carriers (Roselletti et al., 2019).

Another relevant pathway is indeed the nuclear factor kappa-light chain enhancer of activated B cells (NF-κB) response. This response, as well as the production of Dectin-1, results in increased expression of human β-defensin 2 (HBD2), which is seen as a major antimicrobial peptide in the lower genital tract. The amount of HBD2 correlates well with the presence of *Candida* spp. (P < 0.01). In human vaginal epithelial cell lines, the addition of heat-killed *C. albicans* induces expression of Dectin-1 mRNA and NF-κB activation (Kotani et al., 2020).

*An endless list of other experiments investigating the virulence of Candida can be added the those described above, including the influence of the vaginal microbiome, human ions, receptor proteins, and others, but the scope of this work limits the possibility to discuss this topic in further detail. In addition, the expert panel viewed the current information on inflammation and its link to symptomatic disease to be too inconsistent and difficult to implement in clinical practice. Nonetheless, they agreed that further research elucidating the pathogenesis of symptomatic VVC is warranted.*

### The pathogen(s): Species of *Candida*

*C. albicans* and *C. glabrata* are genetically and phenotypically very different (Brunke and Hube, 2013). *C. albicans* is a diploid, polymorphic fungus that exists in three biological phases: yeast, pseudohyphae, and hyphae (Chen et al., 2020). It forms yeast and filament (hyphae and pseudohyphae) forms in response to different conditions and can switch from yeast (blastospore) to hyphal and pseudohyphal growth and back depending on the circumstances. *C. glabrata* is haploid and normally grows only in the yeast form. They differ in their strategies to attach to and to invade the host, to obtain nutrients, and to evade the host immune response and in their outcomes (Brunke and Hube, 2013). Although *C. albicans* follows an aggressive strategy to subvert the host response and to obtain nutrients for its survival, *C. glabrata* uses stealth, evasion, and persistence, without causing severe damage (Brunke and Hube, 2013). However, *C. albicans* and *C. glabrata* are both successful as commensals and as pathogens of humans and can easily adapt their metabolism depending on the available nutrients. Both of them are able to proliferate in either nutrient-rich or nutrient-poor conditions (Van Ende et al., 2019).

For *C.* *albicans* and *C. glabrata*, using diverse sugar sensing systems is crucial for adhesion, oxidative stress resistance, biofilm formation, morphogenesis, invasion, and antifungal drug tolerance, which are determinants of their virulence and resistance to treatments (Van Ende et al., 2019). Fluconazole-resistant *C. albicans* and *C. glabrata* have been detected more often in HIV-positive women and in those with diabetes mellitus compared with otherwise healthy women (Gunther et al., 2014; Zhang et al., 2014). However, no relation has been found between the host glucose metabolism, body mass index, personal or family history of diabetes, and the non-response to maintenance treatment with fluconazole in patients with RVVC (Grinceviciene et al., 2017).

Evidence suggests that asymptomatic colonization, symptomatic acute episodes, and recurrent episodes of vaginal candidosis may be caused by slightly different species of *Candida* (Weissenbacher et al., 2009; Hashemi et al., 2019; Moreira et al., 2021). Indeed, overtime, *Candida* species can develop antifungal drug resistance, with several mechanisms having been identified including overexpression of membrane transporters, altered ergosterol biosynthesis, altered sterol and azole import, genomic variations including loss of heterozygosity and aneuploidy, and chromosomal alterations and rearrangements, among others, that decrease their susceptibility to therapies  (Bhattacharya et al., 2020).

Fluconazole-resistant *C*. *albicans* vaginitis usually represents less than 5% of isolates obtained from women with RVVC (Sobel and Sobel, 2018; Collins et al., 2020). Many fluconazole-resistant *C*. *albicans* strains are also cross-resistant to other azole drugs available for treating VVC. It is therefore essential to obtain susceptibility values of other azole and non-azole antifungal drugs in the diagnostic process (Sobel and Sobel, 2018). Dose-dependent susceptibility and high azole resistance rates have been reported for non-*albicans Candida* species, such as *C*. *glabrata*  (Richter et al., 2005; Ignjatovic et al., 2020) or *C. krusei* (Bitew and Abebaw, 2018).

Among the non-*albicans* species of *Candida*, *C.* *glabrata* is the most common and difficult to predict response to conventional therapy and to eradicate due to its reduced susceptibility to azoles (Kennedy and Sobel, 2010). *C.* *glabrata* has been regarded as a non-pathogenic saprophyte of the normal flora of healthy individuals and is closely related to *Saccharomyces cerevisiae* (Roetzer et al., 2011); it is common in immunocompromised individuals, in older women, and in persons treated with broad-spectrum antibiotics or antifungals (Fidel et al., 1999; Roetzer et al., 2011). *C.* *glabrata* uses the immune evasion strategy to persist in women with VVC and as a pathogenic mechanism (Makanjuola et al., 2018). Its low susceptibility to antifungal agents may explain the increased frequency of colonization (Makanjuola et al., 2018). *C*. *glabrata* has been very commonly observed in HIV-positive women (Zhang et al., 2014) and in those with diabetes mellitus (Gunther et al., 2014).

Likewise, *C.* *krusei* has been reported to cause between 1% and 5% of VVC cases. White women older than 50 years of age and those with perineal lacerations may be at higher risk of infection (Guzel et al., 2013). *C.* *krusei* has been regarded as an intractable cause of RVVC due to its unique and intrinsic resistance to azoles and poor response to other conventional antimycotic agents (Singh et al., 2002; Guzel AB et al., 2013; Makanjuola et al., 2018). The expert panel confirmed that, *in acute VVC episodes, C. albicans is the most frequently encountered organism, whereas in patients with RVVC, especially those on long-term maintenance treatment, non-albicans species are found at higher rates. In addition, several still unknown and unculturable fungal organisms may be present in the vagina of culture-negative symptomatic RVVC patients, as was demonstrated in a study using molecular diagnostic techniques* (Drell et al., 2013).

### The consequences: The clinical, human, and societal burden

RVVC causes physical discomfort affecting the everyday lives of many women (Richter et al., 2005; Moreira et al., 2021), although symptoms from non-*albicans* species are usually less severe than those of C. *albicans*. The common symptoms of C. *albicans* vulvovaginitis are vulval itching, as well as burning, swelling, and redness (Yano et al., 2019), and an increased, non-odorous vaginal discharge (Lines et al., 2020), which will typically become curd-like after a few days. However, discharge may be thin or even completely absent. Itching or painful fissures are often encountered in the interlabial folds, alongside the clitoris and at the ventral perineum. As a result, soreness and introital and vaginal dyspareunia are often present (Woelber et al., 2020). Cyclical symptom patterns are also commonly described, with a typical improvement during menses (Lines et al., 2020).

RVVC presents a significant burden in multiple areas of life. It can negatively affect overall quality of life, mental and physical health, and sexual activity (Zhu et al., 2016; Blostein et al., 2017). In a study of 102 women with RVVC, all health-related quality-of-life dimension scores assessed with the Short-Form Health Survey (SF-36) questionnaire, including physical function, pain, general health, vitality, social functioning, and emotional and mental health, were significantly lower in patients with RVVC compared with healthy women seeking healthcare for other reasons (Zhu et al., 2016).

The overall health status of women with RVVC is lower than the general population both during an acute episode or even outside of infection periods (Aballéa et al., 2013). Women with RVVC are less satisfied with their overall health and report significantly lower or more negative perceptions of their own physical and psychological health and wellbeing than women without RVVC (Fukazawa et al., 2019). In addition, for working women with RVVC, significant productivity losses over a year may occur as around 6 h of work per episode of infection may be missed (Aballéa et al., 2013).

*The panel members’ experience was that their patients with RVVC suffered a high social and emotional burden, which is often not perceived or understood by the public or policy makers. The lead authors recalled at least two patients a year threatening suicide if their RVVC was not resolved, thus asking for understanding and bold therapeutic action. Several panel members had similar patient experiences. Suicidal thoughts do not seem to be mentioned in the literature, but that may be because the subject had never been properly tested in questionnaires.*

### Patients’ perspectives on the disease and on the therapeutic outcomes

Considering the patients’ perspective is an important aspect of understanding the effects of the disease and its treatment on their lives. In a 2016–2018 survey of 284 women, 71% of participants reported needing continual or long-term antifungal therapy to manage their symptoms (Yano et al., 2019). Likewise, other studies found that up to 50% of women with RVVC relapsed after stopping their maintenance therapy regimen (Sobel et al., 2004; Donders et al., 2008a) or that additional fluconazole therapy was continued for longer than 6 months due to either culture-confirmed VVC recurrence, microbiologically unconfirmed but clinically possible VVC recurrence, or due to patients’ preference to continue therapy (Collins et al., 2020). During follow-up, 93.6% of 51 women reported benefits during the maintenance regimen, but 80.9% described relapse after discontinuing the weekly treatment (Collins et al., 2020). A maintenance regimen (denoted the ReCiDiF regimen) has been devised, which allows for the adjustment of the fluconazole dosing frequency when the symptoms, clinical picture, microscopy, and culture findings are all negative, leading to a better tailored, individualized regimen to facilitate a longer duration of treatment at the lowest dose possible for any particular patient (Donders et al., 2008a).

*The expert panel agreed that, for long-term management, maintenance regimens are preferable to episodic treatment in patients with frequent recurrence and a high burden of disease. It was also acknowledged that the frequency of dosing should be kept as low as possible in women using regimens of extended duration. The panel further agreed that, as specialists, they do not underestimate the level of suffering of women with RVVC and that these women need help, and experts will be happy if better and safer treatment is available for the patients.*

### The diagnosis

*Candida* species are opportunistic pathogens that can be present in the vagina without producing symptoms (asymptomatic), can be present and cause sporadic, easy to treat infections (symptomatic episodic VCC), or can cause recurrent symptomatic infections, which may be difficult to treat successfully (RVVC) (Moreira et al., 2021; Sovianti and Devi, 2021). Confirmation of the diagnosis and identification of the *Candida* species are paramount before initiating appropriate long-term treatment. The increase in resistance to existing antifungals and the increasing virulence observed in some species mean that susceptibility testing is required in difficult to cure patients (Hassan et al., 2021; Moreira et al., 2021). In common practice, microscopy and culture are necessary to guide treatment and in cases of clinical non-response. Susceptibility testing *in vitro* and identification of species by PCR can be used to detect infections resistant to antifungals and undetected clinical *Candida*, respectively (Moreira et al., 2021; Sovianti and Devi, 2021). Although detection of *Candida* in asymptomatic patients is not necessarily a significant finding requiring medication, the confirmation of the presence of *Candida* by PCR techniques can be extremely valuable in the management of patients with RVVC who can show a hypersensitivity to even the lowest concentrations of *Candida* antigens (Bernstein and Seidu, 2015; Falsetta et al., 2015). The existence of such hypersensitive reactions to *Candida* antigens, leading to symptomatic disease (Bernstein and Seidu, 2015) and vulvodynia (Falsetta et al., 2015), has extensively been studied and reported in the literature. In addition, PCR-based multilocus sequence techniques serve to identify multiple *Candida* strains from a single sample in persons with and without drug resistance to look for correlations between strains and drug-resistance. (Zhu et al., 2022).

Research evidence suggests that limited awareness of recommended diagnostic practices among healthcare practitioners and a lack of access to point-of-care tools contribute to broad guideline non-adherence (Nyirjesy et al., 2020). Microscopic examination, with the presence of pseudomycelia as a sign of infection and not of colonization, “should be carried out as the first diagnostic step” (Farr et al., 2021a). Likewise, the 2018 European International Union against Sexually Transmitted Infections (IUSTI) World Health Organization (WHO) guideline on the management of vaginal discharge (Sherrard et al., 2018) recommends “that the current best test to diagnose *Candida* in women is microscopy”. The British Association for Sexual Health and HIV recommends that at least two of the episodes should be confirmed by microscopy and culture and that one of these should be a positive culture with moderate or heavy growth of *Candida *sp. to make the diagnosis of RVVC. Full susceptibility testing should also be included in the diagnostic process, if indicated (Saxon et al., 2020). To consolidate the diagnosis of RVVC, the EU guidelines recommend that at least one symptomatic episode is confirmed by culture and species identification (Sherrard et al., 2018; Farr et al., 2021a; McKloud et al., 2021).

*From the perspective of the meeting experts, the diagnostic procedures to detect VVC involve a combination of clinical features and microscopic detection of (pseudo-) hyphae and/or blastospores, supplemented by cultures in unclear cases. Patient history is crucial, as it can reveal former positive diagnostic tests, treatment response, and insight for potential risk factors inducing recurrence.*

*Clinical examination, microscopy of vaginal fluid, and/or cultures should be carried out for differential diagnosis and to rule out other dermatological conditions or infections. In cases of negative mycological findings and persistent skin changes, a vulval skin biopsy may be indicated to rule out dermatoses. However, skin biopsies may not be recommended in cases with a completely normal skin. Microscopic examination using light microscopy or phase contrast microscopy [preferred;* (Donders et al., 2009) *with 400 × optical magnification should be carried out as the first diagnostic step* (Farr et al., 2021a)*]. If symptoms do not resolve, then resistant or non-albicans strains are suspected, or if microscopy is negative, but clinical suspicion of candida is high and needs confirmation, then renewed cultures and antifungal drug sensitivities, and/or PCR should be performed. It has to be acknowledged that cultures and susceptibility testing are expensive and may not be available everywhere, and this is even more true for PCR. Most patients feel confident if physicians use a wet mount in the acute episode, but, unfortunately, its use is not common practice due to a lack of training and habit, and many physicians rely on sending swabs to labs to confirm a diagnosis. As this implies a delay of at least 24–48 h, most women will already be receiving treatment without confirmation that the treatment is the most appropriate. The panel agreed that the results of in vitro resistance testing do not always correlate with clinical practice, so it is the clinical response to treatment, alongside species determination, that is most crucial in therapeutic decisions.*

*In practice, diagnosis and initiation of treatment with fluconazole happen simultaneously to reduce the burden for patients and to contain costs for the healthcare system. As a result, physicians sometimes start treating the patient with only a strong clinical suspicion of RVVC rather than waiting for three or four episodes in 12 months to occur as recommended in guidelines. However, confirmation of the disease with positive tests for the current episode, together with evidence from previous attacks, is strongly recommended.*

**Supplementary Table 1** summarizes similarities and discrepancies that may exist between clinical practice guideline recommendations and practice in the real world.

### The management

#### The patient pathway in the real world

**Figure 1:**
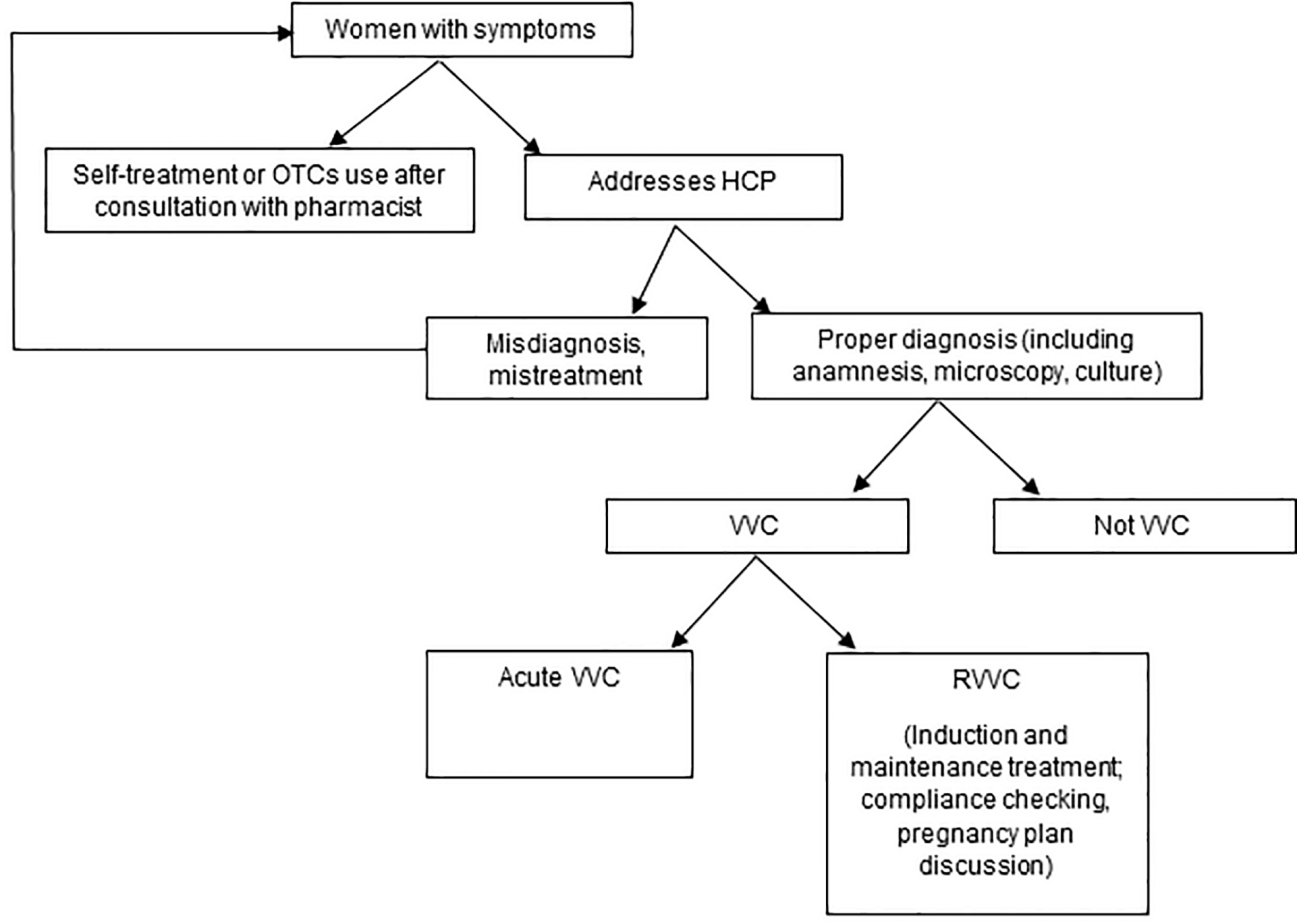
The patient pathway in the real world.

Vaginal infections are an extremely common reason for women to seek advice and care from a physician, pharmacist, or even peer, often leading to over- or misdiagnosis of VVC/RVVC (Sihvo et al., 2000; Lema, 2017). Erroneous self-diagnosis and treatment with antifungals delay proper diagnosis and timely treatment of candidosis, as well as other gynecological conditions (Sihvo et al., 2000). In addition, self-treatment with antifungals may make it more difficult for the healthcare provider to visualize yeast on microscopy, especially when vaginal products have been used (Brown et al., 2021). In the real world, a frequently followed pathway taken by women with RVVC is shown in **Figure 1**.

*The meeting experts agreed that it is difficult to know the frequency of self-treatment without formally researching it. Those who successfully use OTC treatment will not feel the need to come to a healthcare professional. On the other hand, if treatment fails, then women may consult not one but several doctors. Most patients with RVVC were already treated after relating their symptoms to friends, their family, their pharmacist, their general practitioner, and several specialists such as gynecologists, dermatologists, or infectious disease physicians.*

### Pharmacological treatments

#### Efficacy and safety of antifungals recommended in clinical practice guidelines and recurrence rates

Clinical practice guideline recommendations for RVVC are summarized in **Supplementary Table 1**. Induction and maintenance therapies are described. Topical and oral azoles are recommended for induction therapy in some guidelines, whereas oral fluconazole is the first option for maintenance therapy in all guidelines. Second-line maintenance therapies include oral itraconazole or topical clotrimazole. Local boric acid and topical nystatin are options for either induction or maintenance therapies in the Canadian guidelines (Van Schalkwyk et al., 2015). The different mechanisms of action of different antifungals may help to identify therapeutic synergies (Borland et al., 2021). Azoles work by interrupting the conversion of lanosterol to ergosterol and subsequently disrupt fungal membranes. Boric acid inhibits biofilm formation and hyphal transformation of *C.* *albicans*, whereas nystatin works by binding to ergosterol and forming pores in the membrane that lead to K+ leakage, acidification, and disruption of the yeast (**Supplementary Table 2**). Overall, the guidelines recommend topical therapies for mild to moderate symptoms only and advise using oral therapies for severe symptoms and for maintenance to prevent recurrence (Farr et al., 2021a). Not all these treatment modalities are available in all countries.

Although oral fluconazole is highly effective at reducing acute episodes during maintenance therapy, long-term remission after stopping the regimen is only present in about half of patients (Sobel et al., 2004; Donders et al., 2008a). During the treatment phases, relapse rates vary between 21% (Donders et al., 2008a), 57.1% (Sobel et al., 2004), and 71.9% (Bolouri et al., 2009) at 12 months depending upon the characteristics of the therapeutic regimen used, as described in **Supplementary Tables 2** and **3**. Likewise, relapse rates with oral itraconazole reach 47.6% (Fong, 1992), and, with topical clotrimazole, it can be as high as 64% (Fong, 1992) to 84.9% (Roth et al., 1990) at 12 months (**Supplementary Tables 2** and **3**).

The Association of the Scientific Medical Societies in Germany (*Arbeitsgemein-schaft der Wissenschaftlichen Medizinischen Fachgesellschaften:* AWMF) (015/072, level S2k guideline) recommends a dose-reducing suppression therapy based on the ReCiDiF regimen, in which patients are allowed to move to the next level of maintenance only if they are symptom free and microscopy and culture are negative (Donders et al., 2008a; Farr et al., 2021a; Farr et al., 2021b) (**Supplementary Figure 1** and **Supplementary Table 2**).

Likewise, the 2018 European IUSTI/WHO guideline on the management of vaginal discharge recommends an initial intensive regimen of fluconazole 150–200 mg daily for 3 days to attempt mycologic remission before initiating a maintenance regimen (Donders et al., 2008a; Sherrard et al., 2018). Maintenance regimens include oral fluconazole (i.e., 100-, 150-, or 200-mg dose) weekly for 6 months or 200 mg of fluconazole weekly for 2 months, followed by 200 mg biweekly for 4 months, and 200 mg monthly for 6 months, according to the individual response to therapy (Donders et al., 2008a; Sherrard et al., 2018). Clinical guidelines in the UK suggest that it is not clear how the ReCiDiF regimen compares to the standard 6-month regimen proposed by Sobel et al. (Sobel et al., 2004), and therefore, a study comparing these strategies is desirable (Saxon et al., 2020). Other recommendations support a dose-reducing regimen starting with a fluconazole dose of 150 mg weekly for 2–3 months (up to 6 months), which is tapered down to fortnightly for 2 months, and then monthly for 2 months (Sheppard, 2020).

In the ReCiDiF regimen (Donders et al., 2008a), excluding the approximately 10% of women who did not respond to the induction therapy, up to 79% of women were free of disease at 12 months; but at least 40% had been *Candida* positive on microscopy at least once and 50% were recolonized with *Candida* at least once during that year. Maintenance treatment may last differently depending on whether the patient is an optimal responder, partial responder, or non-responder. Overall, adherence to the ReCiDiF regimen was good, and 72% of women were still on the maintenance therapy after 1 year (**Supplementary Tables 2**–**4**).

With the regimen proposed by Sobel et al. (Sobel et al., 2004) (150 mg of oral fluconazole or placebo once a week for 6 months, after a successful induction phase), 42.9% of 387 women were still disease free at 12 months, indicating over half relapsed after maintenance therapy with fluconazole was stopped. Median time to clinical and mycological relapse was 10.2 and 8.4 months, respectively. In another study, the recurrence rate after stopping fluconazole maintenance treatment (Bolouri et al., 2009) was as high as 71.9% (of 64 women with RVVC) at 6 months (**Supplementary Tables 2**-**4**).

Only a Brazilian guideline (Lopes Colombo et al., 2013) and a European guideline (Farr et al., 2021a) include recommendations for fluconazole-resistant *Candida* and vulvovaginitis caused by *C.* non-*albicans* species. Topical boric acid is recommended for RVVC caused by *C glabrata* in the Brazilian guideline, and topical nystatin is a first-line therapy for non*-albicans* and fluconazole-resistant species in the European guideline (**Supplementary Table 2**).

Treatment with first- or second-generation azoles for C *glabrata* infection is usually unsuccessful, and local nystatin, ciclopiroxolamine, or boric acid is recommended (Sobel and Sobel, 2018). According to some, the mycological cure rate may reach 100% (Iavazzo et al., 2011) with topical boric acid. In clinical practice, however, recurrence affects 45.5% (Iavazzo et al., 2011) of patients, and positive cultures for *Candida* have been detected in 56% of patients with RVVC after long-term use of boric acid ovules (Guaschino et al., 2001). However, the European Chemicals Agency issued a warning against the application of boric acid, as there is insufficient data on potential fertility impairment and it may be embryotoxic during pregnancy (Prutting and Cerveny, 1998). Therefore, boric acid can only be considered as a last option, accompanied by contraceptive measures, when it is being prescribed as a magistral formulation in treatment-resistant cases in young, non-pregnant women. The EU guidelines state that the use of boric acid should be limited to “off-label use” for exceptional cases (Farr et al., 2021a).

Topical nystatin appears to be effective for chronic RVVC that is due to non-*albicans* and fluconazole-resistant species (Fan et al., 2015a). The mycological cure rate of RVVC caused by fluconazole-resistant *Candida* species has been reported in 55% (5/9) and 64.3% of patients with a *C.* non-*albicans* species (Fan et al., 2015b). As for locally used azoles, local nystatin is safe for use during pregnancy (**Supplementary Table 2**).

*According to the meeting experts*, *treatment resistance with C. albicans is rare, dose-dependent, and, in common practice, difficult to test. Therefore, the investigation of Candida resistance in clinical practice is uncommon, and there is a need for more reliable, affordable, and readily available tests to check for Candida resistance. There is growing evidence that the number of cases of fluconazole-resistant C. albicans seems to be increasing in clinical practice but is not a substantial problem yet. In addition, in their experience, during maintenance therapy, more non-albicans species and dose-dependent azole susceptible species of C albicans are discovered during relapses than when sporadic VVC is tested. Resistance to azoles, however, is encountered more often and poses a big challenge when it occurs.*

#### Commonly used pharmacological treatments in clinical practice

Overall, treatments that are commonly used in clinical practice are aligned to guideline recommendations. Oral fluconazole is the first-line drug of choice followed by OTC azoles and other OTC drugs, such as boric acid and probiotics, in second and third line, respectively. Probiotics, defined as live microorganisms, which, when administered in adequate amounts, confer a health benefit on the host (Falagas et al., 2006), and hygiene products are in fourth place of choice and are the least frequently recommended to treat RVVC. There is no evidence nor are there any estimates on how often a switch from one OTC product to another happens, but in clinical practice, it is conceived as being rather frequent. Potential reasons for switching include ineffectiveness or side effects of treatment, incorrect diagnosis, patients’ despair and distress, or misinformation. Although not recommended, self-treatment may happen with women who then go back to the healthcare professional seeking proper care after treatment failures (Mårdh et al., 2004). Personal (attitudes, beliefs, values, knowledge and experience, and emotions), environmental (culture, social networks and norms, media, and life context), and behavioral factors are among the primary reasons that influence women’s decisions to self-treat vaginal symptoms (Mårdh et al., 2004; Theroux, 2005)

Evidence shows that physician-treated cases achieved a higher level of symptom relief (84%) than self-medicated cases (57%) (Yano et al., 2019). The gynecologist is the main RRVC treatment-prescribing specialist in most European countries. Other healthcare professionals may also be involved, such as the general practitioner and family physicians, internists, dermatologists, urologists, and the sexual health clinician (UK), and midwifes may give advice in The Netherlands, Italy, or Spain. It is crucial to ensure the necessary level of knowledge on RVCC treatment among all these groups of healthcare professionals, especially among those with less expertise. Gynecologists and trainees may also have unmet training needs in the optimal assessment and management of RVVC (Borland et al., 2021).

Idiopathic non-responders, relapse, resistance, inadequate prophylaxis, and women’s choices, habits, and behaviors challenge the success of RVVC treatment (Lema, 2017). Rapid onset of symptom relief, safety compared with already known medications, simple therapy schedules, avoidance of drugs metabolized by the liver, and resistance to or failure on fluconazole may drive women’s treatment preferences (Martens, 2018). As part of the treatment plan in women with an altered glucose tolerance, lowering glycemia by the reducing the intake of sugars or by glucose lowering medication may be important (Donders et al., 2011), whereas pregnancy anticipation in women of childbearing age is of relevance in the self-management of RVVC.

*All meeting experts agreed that using an azole therapy is the most-favored first-line treatment. However*, g*ynecologists may deviate from the recommended guidelines for two reasons: because of a fear of side effects (e.g., liver damage by fluconazole) and because treatments are too expensive for patients. Furthermore, experts discussed that physicians may be reluctant to recommend topical treatments in recurrent cases to avoid complicating sample taking and microscopic examination.*

*Although fluconazole and itraconazole remain good products for acute episodes, even in women with frequent relapses, an estimated 20%–50% of patients still claim only short symptomatic relief. Despite efficacy during acute episodes, consolidation by preventive maintenance is often warranted. Some patients require long-term treatment for several years to prevent relapses. In about 30% of women, multiple recurrences occur despite close follow-up and dose adjustments during regimens such as ReCiDiF. In general, the treatment is well tolerated, but, occasionally, patients may experience side effects (e.g., skin problems, hair loss, dizziness, and stomach problems).*

*In patients with RVVC, the treatment will be different depending on whether it is for C. albicans or non-albicans infection. In the latter case, it is of no use to try azole therapy. The assembled experts agreed that the introduction of alternative drugs to oral fluconazole for the treatment of women with RVVC would be also interesting in cases of dose-dependent antifungal resistance, which is associated with a need for ever increasing doses of classical azoles or a switch to treatments that may be difficult to obtain or are expensive. Similarly, in cases of C. glabrata infection, RVVC, and diabetes and in the case of no-response to ReCiDiF, efficient and affordable alternative treatments would be very welcome.*

*The meeting experts agreed that the benefits and disadvantages of simultaneous contraception for a certain period should be discussed with patients. In their view, pregnancy plans should not be a barrier to oral azole treatment and patients should be well informed. It is preferable that pregnancy does not occur during treatment respecting the half-life of the medication. If patients agree with contraception for the defined period, there should be no resistance to treatment of cases where other treatments have failed. Overall, patients would use treatment for as long as it is prescribed or recommended by their healthcare practitioner.*

### Treatment duration in clinical practice guidelines and in the real world

According to clinical practice guidelines, treatment of RVVC should include an induction followed by a maintenance period that should last for at least 6 months to prevent recurrence (Sherrard et al., 2018; Saxon et al., 2020; Farr et al., 2021a) (**Supplementary Figure 2**). However, there are no trials addressing the optimal duration of suppressive therapy as the majority of trials have used 6 months maintenance as standard (Saxon et al., 2020). It is recommended that, if recurrence after a maintenance regimen is infrequent, then each episode should be treated independently, whereas if recurrent disease is re-established, then the induction and maintenance regimens should be repeated, prolonged, or adjusted (Saxon et al., 2020).

In clinical practice (**Supplementary Table 1**), experts in the meetings agreed that *treatment may last for 3 to 6 months, but with 50% to 70% of women relapsing after stopping maintenance*. *Often, maintenance therapy continues beyond 6 months, even years in some problematic RVVC cases; this is not so uncommon in clinical practice. In the ReCiDiF regimen, the dosing frequency is adjusted and decreases when symptoms are well controlled. This implies the regimen lasts for 1 year for optimal responders, after which they can stop the treatment. For “suboptimal responders” the regimen is adjusted and may last longer. Some patients require one tablet a month or one tablet every 2 weeks to be continued for a prolonged period, depending on their relapse and response rates during the regimen.*

### Role of probiotics for RVVC

In the view of experts, in the light of the frequent coexistence in the human vagina of *Candida* with lactobacilli, the use of lactobacilli as probiotics may not be logical. Still, many companies claim that their probiotic strains are efficient in preventing or curing *Candida* infections. However, often, these products were tested in laboratories but seldomly in human patients. One of the authors of this paper (GD) tested lactobacilli specifically selected for their epithelial cell adherence qualities, yeast biofilm reducing capacity, and antifungal action in women with acute symptomatic VVC. The author found that, in 45% of the cases, no rescue medication was needed (Donders et al., 2020) and, in patients with improvement with probiotics, lactobacilli were increased and fungi decreased as compared with women having received rescue medication with fluconazole (Oerlemans et al., 2020).

Two comprehensive reviews (Xie et al., 2013; Shenoy and Gottlieb, 2019) on the effects of probiotics against symptomatic VVC have highlighted the scarce and low-quality evidence, with a need for robust randomized trials to ascertain whether probiotics can have a beneficial effect on the (adjuvant) treatment of acute VVC or in the prevention of recurrences in patients with RVVC.

## Discussion

Overall, RVVC is a highly burdensome, long-lasting medical condition that heavily compromises the activities of women and their quality of life. Women are at risk of RVVC at all ages, but their fertile period entails the highest risk. RVVC is challenging for both the patient and her healthcare professional. Clinical practice guidelines consistently recommend the use of oral fluconazole as first choice, but this proves insufficient for resistant *C. albicans* and non-*albicans* species. In addition, the literature suggests that RVVC can frequently feature culture negative symptomatic episodes. Negative cultures may be reflective of recent treatment, swab quality, and sampled location among other possible factors, for instance, misdiagnosis (the itching and burning of vulvodynia, *Lichen sclerosus*, and so on). Thus, the guidelines that focus only on a positive culture may not provide optimal care options for all patients, especially for those experiencing frequent recurrences (Bradfield Strydom et al., 2021). Adherence and persistence can be well accepted if RVVC is comprehensively explained. Patient education and engagement in treatment decision-making are of great importance.

Key to the successful treatment of RVVC is making the right diagnosis, recommending the right treatment, adequately informing and educating the patients, and seeking appropriate adherence to and persistence with the therapy. After regimen failure, there is need for a choice of treatment in RVVC. Well-tolerated affordable new drugs, with an optimized mechanism of action, apart from the currently available azoles, with long-lasting efficacy ideally not only against therapy resistant *C.* *albicans* but also against non-*albicans* species and with a favorable safety profile, would be very welcome. **Supplementary Table 4** summarizes current developments.

A potential treatment option in the future might be oteseconazole, which is an oral selective inhibitor of fungal lanosterol demethylase (CYP51A1), the targeted mechanism of which specifically minimizes safety issues and limitations of efficacy (Farr et al., 2021a). Oteseconazole showed strong activity against azole-resistant *C. albicans* and non-*albicans* species, such as *C. glabrata* and *C*. *krusei*, in the treatment of chronic RVVC, with affected patients showing no recurrence for a duration of 48 weeks (Brand et al., 2018). Oteseconazole has the potential for fewer adverse events than are seen with previous-generation azole antifungals. Potent efficacy and safety data support the potential value of oteseconazole to treat both azole-sensitive and azole-resistant *C. albicans* and non-*albicans* strains in women who cannot be treated in other ways (Sobel and Nyirjesy, 2021; Sobel JD/Donders G et al., 2022).

Other novel antifungal agents are in development. For instance, ibrexafungerp is an oral β-1,3-D-glucan synthase inhibitor (GSI) of the enfumafungin-derived triterpenoid class of antifungals (echinocandins) (Gamal et al., 2021). GSIs are a group of drugs that act by inhibition of β-1,3-D-glucan synthase, a key enzyme in the biosynthesis of ß-(1,3)-D-glucan, a major component of the fungal cell wall. Preclinical studies have shown equal or superior *in vitro* activity compared with echinocandins, such as caspofungin and micafungin, against both wild-type and echinocandin-resistant *C.* *glabrata* isolates (Gamal et al., 2021).

As another way to improve treatment, topical miconazole has been combined with the quaternary ammonium compound domiphen bromide that acts as a miconazole potentiator against *Candida *biofilms. *In vitro* and *in vivo* preliminary evidence has indicated that the development of resistance against the combination did not occur and has thus highlighted the potential for this combination therapy to treat *Candida* biofilm-related infections (Tits et al., 2021).

An immunotherapeutic vaccine (NDV-3A) containing a recombinant *C.* *albicans* adhesin/invasin protein has been evaluated for the prevention of RVVC in an exploratory randomized, double-blind, placebo-controlled, phase 2 trial (Edwards et al., 2018). One intramuscular single dose of NDV-3A was safe, generated rapid and robust B- and T-cell immune responses, and reduced the frequency of symptomatic episodes of RVVC for up to 12 months in women aged <40 years. Additional preclinical and clinical studies are required to further confirm the efficacy and safety of these novel therapies in treating fungal infections causing RVVC.

In the view of the panel of experts, future research should unravel why some patients are more sensitive to the presence of yeast, whereas others, with signs of fulminant infection, do not seem to be troubled by it. Especially for patients with RVVC, the triggers of symptomatic disease must be better explored. Currently, with the help of Gedeon-Richter, the International Society for Infectious Diseases in Obstetrics and Gynecology is designing a questionnaire specific for exploring the consequences of (recurrent) VVC, with an emphasis on quality of life and sex life. Further, new drugs are badly needed to be developed, as described earlier in the paper. For decades, no significant improvements have been made in the current therapy based on azoles. One new potent azole with a very long half-life, oteseconazole, and some new non-azole drugs, such as ibrexafungerp, need to be studied in clinical trials to determine their optimal dosing regimen, especially in patients with RVVC who are refractory to current maintenance regimens, such as ReCiDiF. Finally, adjuvant therapies like probiotics with anti-*Candida* activity and vaccines decreasing the symptoms and burden of RVVC may be valued additions to the treatment armamentarium.

## Author contributions

GD and RC contributed to conception and design of the study; all authors contributed to the acquisition of data and participated in the meetings and discussion rounds; GD, JP, IS, PH, FS, WM, JB, and JK critically reviewed drafts of the manuscript; GD critically reviewed the final version of the manuscript; all authors approved the submitted version of the manuscript.

## Funding

Gedeon Richter Plc., Hungary funded the project.

## Acknowledgments

The authors thank Silvia Paz Ruiz MD MMedSci (Terminal 4 Communications) for providing medical writing support and Maren White BA (Terminal 4 Communications) for editorial support in accordance with the Good Publication Practice (GPP3) guidelines (http://www.ismpp.org/gpp3).

## Conflict of interest 

GD, IS, JP, PH, FS, JB, JK, JV, BS, and WM are expert consultants to the pharmaceutical industry, including Gedeon Richter. RC is an employee at Gedeon Richter.This study received funding from Gedeon Richter Plc. The funder had the following involvement with the study: design and collection of data, and on the decision to submit the manuscript for publication. Authors led the analysis and interpretation of data, and the writing of the manuscript. All authors declare no other competing interests.

## Publisher’s note

All claims expressed in this article are solely those of the authors and do not necessarily represent those of their affiliated organizations, or those of the publisher, the editors and the reviewers. Any product that may be evaluated in this article, or claim that may be made by its manufacturer, is not guaranteed or endorsed by the publisher.

## Disclaimer

All claims expressed in this article are solely those of the authors and do not necessarily represent those of their affiliated organizations or those of the publisher, the editors, and the reviewers.

## Supplementary material

Supplementary Figure 1 European clinical practice recommendations to treat chronic RVVC based on the ReCiDif decreasing dose regimen.

Supplementary Figure 2 Recurrent vulvovaginal candidosis diagnosis and treatment flows according to European guideline recommendations.

Supplementary Table 1 Clinical practice in the real world compared to guideline recommendations.

Supplementary Table 2 Summary of clinical practice guideline recommendations for the management of vulvovaginal candidosis and recurrent vulvovaginal candidosis.

Supplementary Table 3 Mechanism of action, safety and indication / contraindication of current antifungals used for RVVC.

Supplementary Table 4 Disease-free, relapse, failure, and patient adherence, persistence and satisfaction with published treatment regimens for recurrent vulvovaginal candidosis.

Supplementary Table 5 Investigational drugs: summary of mechanism of action and preliminary results.
