# Supplementary figures and images for: Management of recurrent vulvovaginal candidosis: Narrative review of the literature and European expert panel opinion
Source: Front Cell Infect Microbiol. 2022 Sep 9;12:934353. doi: 10.3389/fcimb.2022.934353 (PMC9504472; doi:10.3389/fcimb.2022.934353)

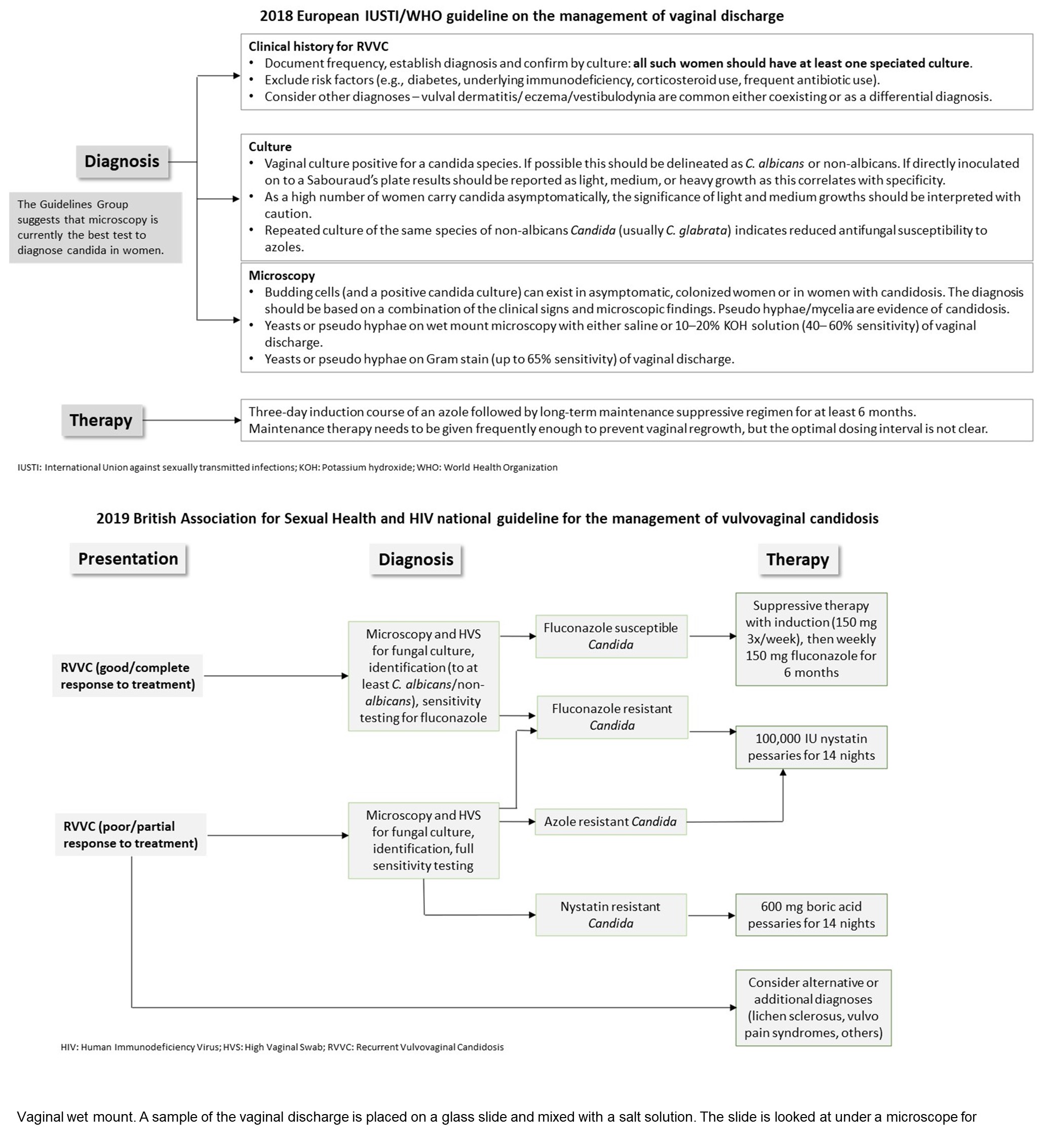

Supplement: Supplementary Figure 1 — European clinical practice recommendations to treat chronic RVVC based on the ReCiDif decreasing dose regimen. [file Image_1.jpeg]

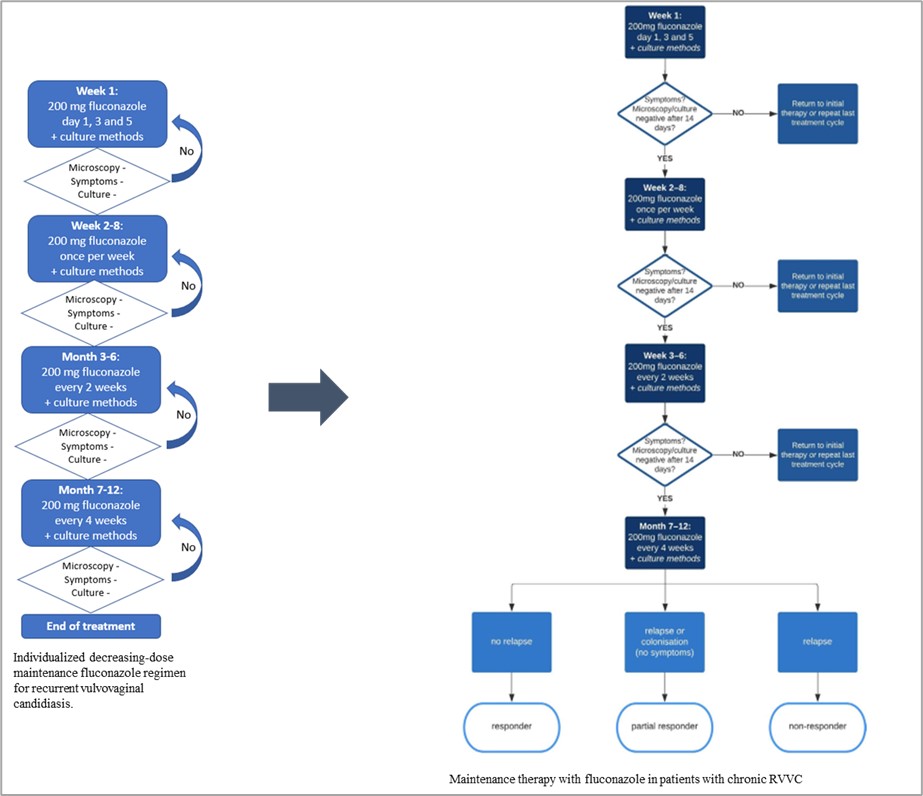

Supplement: Supplementary Figure 2 — Recurrent vulvovaginal candidosis diagnosis and treatment flows according to European guideline recommendations. [file Image_2.jpeg]
