# Supplementary material for: Management of recurrent vulvovaginal candidosis: Narrative review of the literature and European expert panel opinion
Source: Front Cell Infect Microbiol. 2022 Sep 9;12:934353. doi: 10.3389/fcimb.2022.934353 (PMC9504472; doi:10.3389/fcimb.2022.934353)
Supplement: Supplementary Table 1 — Clinical practice in the real world compared to guideline recommendations. [file Table_1.docx]

**SUPPLEMENTARY Table 1. clinical practice in the real world compared to guideline recommendations**

|  | Common clinical practice based on the narrative of the panel experts | British Association for Sexual Health and HIV (BASHH) (2019) (1) | 2018 European IUSTI / WHO guidelines on the management of vaginal discharge (2) | The Societies of Gynaecology and Obstetrics of Germany, Austria, and Switzerland (2021) (3) (4) |
| --- | --- | --- | --- | --- |
| Diagnosis | *‘Cultures are expensive and not easily available, so gynecologists very seldom use them.’*  *‘If symptoms don't resolve, we would then do culture and sensitivity and consider alternative agents.’*  *‘I do cultures if I suspect resistance, non-albicans strain or if microscopy is negative.’*  *‘Initial diagnosis and starting treatment with fluconazole frequently happen without culture.’* | Culture + appropriate speciation and sensitivity testing depending on clinical indication. | Current best test to diagnose *candida* in women is microscopy. | Combination of clinical features + microscopic detection of (pseudo‐) hyphae + cultural methods in unclear cases.  Treatment for vulvovaginal candidosis should always follow proper diagnostic work‐up based on medical anamnesis, symptoms, microscopy and, in some cases, culture methods. |
| Treatment | Treatment regimens may last for 3 to 6 months in most cases  *‘The ReCiDiF regimen lasts for 1 year. Good responders only take one tablet per month. For 'suboptimal responders' the regimen is adjusted and may last longer. Some patients require one tablet a month or one tablet every 2 weeks continued for a prolonged period.’* | Non-azole therapies to be reserved for azole resistance and certain non-*albicans* *Candida* species.  Induction regimen to ensure clinical remission, followed immediately by a maintenance regimen: fluconazole 150 mg every 72 hours for 3 doses followed by 150 mg weekly for a 6-month period. | Current best treatment for persistent and recurrent *candida* in women: three-day induction course of an azole + long-term maintenance suppressive regimen for at least six months. | Long‐term antifungal treatments can be used for chronic recurrent vulvovaginal candidosis, using various regimens with little evidence: (a) 150 mg fluconazole weekly for 6 months, (b) 200 mg fluconazole for 3 days in the first week, followed by a maintenance regimen once the patient is free of symptoms or fungi with 200 mg fluconazole once per month for 12 months (decreasing dose if good response). |
| IUSTI, International Union against sexually transmitted infections; WHO, World Health Organization | | | | |

References

1. Saxon C, Edwards A, Rautemaa-Richardson R, Owen C, Nathan B, Palmer B, et al. British Association for Sexual Health and HIV national guideline for the management of vulvovaginal candidiasis (2019). Int J STD AIDS. 2020;31(12):1124–44.

2. Sherrard JE (IUSTI/WHO) IU against sexually transm, Wilson J, Donders G, Mendling W, Jensen JS. 2018 European (IUSTI/WHO) International Union against sexually transmitted infections (IUSTI) World Health Organisation (WHO) guideline on the management of vaginal discharge. Int J STD AIDS. 2018;29(13):1258–72.

3. Farr A, Effendy I, Frey Tirri B, Hof H, Mayser P, Petricevic L, et al. Guideline: Vulvovaginal candidosis (AWMF 015/072, level S2k). Mycoses. 2021;64(6):583–602.

4. Farr A, Effendy I, Tirri BF, Hof H, Mayser P, Petricevic L, et al. Vulvovaginal Candidosis (Excluding Mucocutaneous Candidosis): Guideline of the German (DGGG), Austrian (OEGGG) and Swiss (SGGG) Society of Gynecology and Obstetrics (S2k-Level, AWMF Registry Number 015/072, September 2020). Geburtshilfe Frauenheilkd [Internet]. 2021 Apr 1 [cited 2022 Aug 1];81(4):398. Available from: /pmc/articles/PMC8046514/
