# Supplementary material for: Management of recurrent vulvovaginal candidosis: Narrative review of the literature and European expert panel opinion
Source: Front Cell Infect Microbiol. 2022 Sep 9;12:934353. doi: 10.3389/fcimb.2022.934353 (PMC9504472; doi:10.3389/fcimb.2022.934353)
Supplement: Supplementary Table 2 — Summary of clinical practice guideline recommendations for the management of vulvovaginal candidosis and recurrent vulvovaginal candidosis. [file Table_2.docx]

**SuplemEntary Table 2. Summary of clinical PRACTICE guideline Recommendations for the management of vulvovaginal candidosis**

**and recurrent vulvovaginal candidosis**

| Scientific Society, Country, year (Ref) | Recommended therapy | | | Regimen | Strength of recommendation and quality of evidence^a^ |
| --- | --- | --- | --- | --- | --- |
| Latin America | | | | | |
| Brazilian guidelines for the management of candidiasis (2013)(1) | Induction therapy | First-line therapy | Topical imidazole formulation  Oral fluconazole | 7-14 days  150 mg/day every 72h (days 1, 4, and 7) | A-I  A-II |
|  | Maintenance therapy | First-line therapy | Oral fluconazole | 150 mg/day once a week for six months | A-II |
|  |  | Second-line therapy | Clotrimazole  Itraconazole | 500 mg suppositories twice a week  200 mg twice a week | B-I  B-I |
|  | RVVC caused by *Candida glabrata* | First-line therapy | Boric acid suppositories | 600 mg/day for 14 days | B-I |
| NORTH AMERICA | | | | | |
| Infectious Diseases Society of America (IDSA)  United states (2016) (2) | Induction therapy | First-line therapy^b^ | Topical agent or oral fluconazole | 10-14 days | Strong recommendation; high-quality evidence |
|  | Maintenance therapy | First-line therapy^b^ | Oral fluconazole | 150 mg weekly for 6 months | Strong recommendation; high-quality evidence |
| Centers for Disease Control and Prevention  United states (2021) | Induction therapy | First-line therapy | Oral fluconazole | 100, 150 or 200 mg oral dose every third day for a total of 3 doses | Not reported |
|  |  | Second-line therapy | Topical therapy | 7-14 days of topical therapy | Not reported |
|  | Maintenance therapy | First-line therapy^b^ | Oral fluconazole | 100-mg, 150-mg, or 200-mg dose weekly for 6 months | Not reported |
| The Society of Obstetricians and GynaecologistS of Canada (2015) (3) | Induction therapy^c^ |  | Imidazole cream  Oral fluconazole  Boric acid insert  Clotrimazole insert | 10-14 days  150 mg: 3 doses, 72 hours apart  300 to 600 mg daily × 14 days  500 mg: once monthly × 6 months | Not reported  II-2A  Not reported  Not reported |
|  | Maintenance therapy^c^ |  | Oral fluconazole  Boric acid insert  Oral ketoconazole | 150 mg weekly for 6 months  300 mg daily × 5 days at the beginning of each menstrual cycle  100 mg once daily | II-2A  Not reported  Not reported |
| Europe | | | | | |
| International Union against sexually transmitted infections (IUSTI) World Health Organisation (WHO) (2018) (4) | Induction therapy | First-line therapy | Oral fluconazole | 150 mg/day every 72h (days 1, 4 and 7) | 2C |
|  |  |  |  | 200 mg/day every 72h (days 1, 4 and 7) | 2C |
|  | Maintenance therapy | First-line therapy | Oral fluconazole | 150 mg weekly for six months | 2C |
|  |  |  |  | 200 mg fluconazole weekly for two months, followed by 200 mg biweekly for four months, and 200 mg monthly for six months, according to the individual response to therapy |  |
| The Societies of Gynaecology and Obstetrics of Germany, Austria, and Switzerland^d^ (2021) (5) (6) | Induction therapy | First-line therapy^b^ | Oral fluconazole | 200 mg/day three days per week (for one week) | The primary therapy for chronic RVVC should be oral therapy, possibly in the form of long-term suppressive therapy: level of expert consensus +++ (strong consensus) |
|  | Maintenance therapy | First-line therapy^b^ | Oral fluconazole | 200 mg oral fluconazole per month for a period of one year as follows: 200 mg oral fluconazole once a week (week 2-8); 200 mg oral fluconazole every two weeks (months 3-6); 200 mg oral fluconazole every four weeks (months 7-12) |  |
|  |  | First-line therapy (non*-albicans* and fluconazole-resistant species) ^b^ | Topical nystatin | If *C. krusei*, first, topical clotrimazole for 2 weeks followed by ciclopirox or nystatin | Not reported |
| British Association for Sexual Health and HIV (BASHH)  United Kingdom (2019) (7) | Induction therapy | First-line therapy | Oral fluconazole | 150 mg/day each 72h (days 1, 4, and 7) | 1A |
|  |  | Second-line therapy | Topical imidazole | 7-14 days | 2C |
|  | Maintenance therapy | First-line therapy | Oral fluconazole | 150 mg weekly for 6 months | 1A |
|  |  | Second-line therapy | Clotrimazole pessary  Itraconazole | 500 mg intravaginally once a  week for 6 months  50-100 mg orally daily for 6 months | 1B  2C |
| OCEANIA | | | | | |
| australasian sexual health alliance australia (2018) (8) (9) | Induction therapy | First-line therapy | Azole cream  Oral fluconazole | Long course  150 mg: 3 doses, 72 hours apart | Not reported  Not reported |
|  |  | Second-line therapy | Oral itraconazole | 100 mg daily until asymptomatic | Not reported |
|  | Maintenance therapy | First-line therapy | Oral fluconazole | 150 mg weekly for 6 months | Not reported |
|  |  | Second-line therapy | Oral itraconazole | 100 mg weekly for 6 months | Not reported |
| ^a^ Scientific Societies adopt different criteria to establish the quality of the evidence and the strength of recommendations. Overall, the earlier the letter in the alphabet and the lower the accompanying number, the better-quality evidence and stronger recommendation  ^b^ Recommendation for second-line therapy was not reported  ^c^ Recommendations were not divided into first- and second-line therapy  ^d^ DGGG: Deutsche Gesellschaft für Gynaekologie und Geburtshilfe, OEGGG: Österreichische Gesellschaft für Gynäkologie und Geburtshilfe, SGGG: Schweizerische Gesellschaft für Gynäkologie und Geburtshilfe | | | | | |

References

1. Lopes Colombo A, Guimarães T, Camargo LFA, Richtmann R, de Queiroz-Telles F, Salles MJC, et al. Brazilian guidelines for the management of candidiasis - a joint meeting report of three medical societies: Sociedade Brasileira de Infectologia, Sociedade Paulista de Infectologia and Sociedade Brasileira de Medicina Tropical. Brazilian J Infect Dis. 2013 May;17(3):283–312.

2. Pappas PG, Kauffman CA, Andes DR, Clancy CJ, Marr KA, Ostrosky-Zeichner L, et al. Clinical Practice Guideline for the Management of Candidiasis: 2016 Update by the Infectious Diseases Society of America. Clin Infect Dis. 2015;62(4):e1–50.

3. Van Schalkwyk J, Yudin MH, Allen V, Bouchard C, Boucher M, Boucoiran I, et al. Vulvovaginitis: Screening for and Management of Trichomoniasis, Vulvovaginal Candidiasis, and Bacterial Vaginosis. J Obstet Gynaecol Canada. 2015;37(3):266–74.

4. Sherrard JE (IUSTI/WHO) IU against sexually transm, Wilson J, Donders G, Mendling W, Jensen JS. 2018 European (IUSTI/WHO) International Union against sexually transmitted infections (IUSTI) World Health Organisation (WHO) guideline on the management of vaginal discharge. Int J STD AIDS. 2018;29(13):1258–72.

5. Farr A, Effendy I, Frey Tirri B, Hof H, Mayser P, Petricevic L, et al. Guideline: Vulvovaginal candidosis (AWMF 015/072, level S2k). Mycoses. 2021;64(6):583–602.

6. Farr A, Effendy I, Tirri BF, Hof H, Mayser P, Petricevic L, et al. Vulvovaginal Candidosis (Excluding Mucocutaneous Candidosis): Guideline of the German (DGGG), Austrian (OEGGG) and Swiss (SGGG) Society of Gynecology and Obstetrics (S2k-Level, AWMF Registry Number 015/072, September 2020). Geburtshilfe Frauenheilkd [Internet]. 2021 Apr 1 [cited 2022 Aug 1];81(4):398. Available from: /pmc/articles/PMC8046514/

7. Saxon C, Edwards A, Rautemaa-Richardson R, Owen C, Nathan B, Palmer B, et al. British Association for Sexual Health and HIV national guideline for the management of vulvovaginal candidiasis (2019). Int J STD AIDS. 2020;31(12):1124–44.

8. Bradfield Strydom M, Walpola RL, Khan S, Ware RS, Tiralongo E. Evidence-based update on Australasian pharmaceutical prescribing approaches for recurrent vulvovaginal candidiasis. Aust New Zeal J Obstet Gynaecol. 2021;61(4):496–9.

9. Candidiasis - Australian STI Management Guidelines [Internet]. [cited 2021 Oct 4]. Available from: http://www.sti.guidelines.org.au/sexually-transmissible-infections/infections-associated-with-sex/candidiasis#management
