# Supplementary material for: Management of recurrent vulvovaginal candidosis: Narrative review of the literature and European expert panel opinion
Source: Front Cell Infect Microbiol. 2022 Sep 9;12:934353. doi: 10.3389/fcimb.2022.934353 (PMC9504472; doi:10.3389/fcimb.2022.934353)
Supplement: Supplementary Table 3 — Mechanism of action, safety and indication / contraindication of current antifungals used for RVVC. [file Table_3.docx]

**sUPLEMENTARY Table 3. MECHANISM OF ACTION, safety AND INDICATION/CONTRAINDICATION of current antifungaLs used for recurrent vulvovaginal candidosis**

| Agent | Mechanism of action | Safety | Indication/Contraindication during pregnancy |
| --- | --- | --- | --- |
| Oral treatment | | | |
| fluconazole  (1) (2) (3) (4) | Interruption of the conversion of lanosterol to ergosterol via binding and inhibiting fungal cytochrome P-450- dependent enzyme lanosterol 14-α-demethylase and subsequent disruption of fungal membranes | Well tolerated and free of serious adverse effects  Rare adverse events:   - Headaches - Dizziness - Minor abdominal discomfort | **Contraindicated during pregnancy** (5):   - Any dose of oral fluconazole while pregnant may be associated with a higher chance of miscarriage - Taking higher doses of fluconazole over 150 mg in early pregnancy may be linked to a higher chance of a newborn with a heart defect - FDA labels fluconazole 150 mg category C*** |
| itraconazole  (6) (3) (7) |  | - Local allergic reaction led to discontinuations in two patients | **Contraindicated during pregnancy:**   - Itraconazole capsules must not be used during pregnancy (except for life-threatening cases). - Women of childbearing potential taking itraconazole should use contraceptive precautions. Effective contraception should be continued until the menstrual period following the end of itraconazole therapy. - FDA labels itraconazole category C*** |
| Ketoconazole (8) | Oral formulations for the treatment of fungal infections were suspended the European Medicines Agency in July 2013 because of the risk of liver injury | | |
| Topical treatment | | | |
| clotrimazol  (9) (10) (11) | Interruption of the conversion of lanosterol to ergosterol via binding and inhibiting fungal cytochrome P-450 dependent enzyme lanosterol 14-α-demethylase and subsequent disruption of fungal membranes | No reported | **Can be used during pregnancy**, but only under the supervision of a physician or midwife.  At the low systemic exposures of clotrimazole following vaginal treatment, harmful effects with respect to reproductive toxicity are not predicted.  FDA pregnancy category B*** |
|  |  |  |  |
|  |  | No reported |  |
|  |  |  |  |
| boric acid  (12) (13) (3) | Inhibition of biofilm formation and hyphal transformation of *Candida* albicans | - Vaginal burning sensation - Slight water discharge during treatment - Vaginal erythema | The European Chemicals Agency issued a warning against the application of boric acid, as it can impair fertility and might be embryotoxic (14) |
|  |  |  |  |
| nystatin  (15) (7) (3) | Binding to ergosterol, a major component of the fungal cell membrane. When present in sufficient concentrations, it forms pores in the membrane that lead to K+ leakage, acidification, and death of *Candida* | - Vaginal burning pain - Vulvar oedema and vulvar itching | Nystatin vaginal tablets have been assigned to pregnancy category A by the FDA***. Nystatin topical is only recommended for use during pregnancy when benefit outweighs risk. |
| ***FDA Pregnancy Categories  Category A  Adequate and well-controlled studies have failed to demonstrate a risk to the fetus in the first trimester of pregnancy (and there is no evidence of risk in later trimesters).  Category B  Animal reproduction studies have failed to demonstrate a risk to the foetus and there are no adequate and well-controlled studies in pregnant women.  Category C  Animal reproduction studies have shown an adverse effect on the fetus and there are no adequate and well-controlled studies in humans, but potential benefits may warrant use of the drug in pregnant women despite potential risks.  Category D  There is positive evidence of human fetal risk based on adverse reaction data from investigational or marketing experience or studies in humans, but potential benefits may warrant use of the drug in pregnant women despite potential risks.  Category X  Studies in animals or humans have demonstrated fetal abnormalities and/or there is positive evidence of human fetal risk based on adverse reaction data from investigational or marketing experience, and the risks involved in use of the drug in pregnant women clearly outweigh potential benefits.  FDA, Food and Drug Administration; RVVC, recurrent vulvovaginal candidosis | | | |

References

1. Sobel JD, Wiesenfeld HC, Martens M, Danna P, Hooton TM, Rompalo A, et al. Maintenance Fluconazole Therapy for Recurrent Vulvovaginal Candidiasis. N Engl J Med. 2004 Oct;351(9):876–83.

2. EMA. Assessment report pursuant to Article 30 of Directive 2001 / 83 /EC, as amended. Diflucan and associated names. [Internet]. Patient Health Protection. 2021 [cited 2021 Dec 6]. Available from: https://www.ema.europa.eu/en/documents/referral/diflucan-article-30-referral-assessment-report_en.pdf

3. Mycovia Pharmaceuticals Submits New Drug Application to the U.S. FDA for Oteseconazole for the Treatment of Recurrent Vulvovaginal Candidiasis | Business Wire.

4. Pasko MT, Piscitelli SC, Van Slooten AD. Fluconazole: A new triazole antifungal agent. DICP, Ann Pharmacother. 1990;24(9):860–7.

5. Zhang Z, Zhang X, Zhou YY, Jiang CM, Jiang HY. The safety of oral fluconazole during the first trimester of pregnancy: a systematic review and meta-analysis. BJOG An Int J Obstet Gynaecol. 2019 Dec;126(13):1546–52.

6. Witt A, Kaufmann U, Bitschnau M, Tempfer C, Özbal A, Haytouglu E, et al. Monthly itraconazole versus classic homeopathy for the treatment of recurrent vulvovaginal candidiasis: A randomised trial. BJOG An Int J Obstet Gynaecol. 2009 Oct;116(11):1499–505.

7. Pilmis B, Jullien V, Sobel J, Lecuit M, Lortholary O, Charlier C. Antifungal drugs during pregnancy: An updated review. J Antimicrob Chemother. 2015 Jan 1;70(1):14–22.

8. EMA. European Medicines Agency recommends suspension of marketing authorisations for oral ketoconazole | European Medicines Agency.

9. Roth AC, Milsom I, Forssman L, Wåhlén P. Intermittent prophylactic treatment of recurrent vaginal candidiasis by postmenstrual application of a 500 mg clotrimazole vaginal tablet. Sex Transm Infect. 1990 Oct 1;66(5):357–60.

10. Sobel JD, Schmitt C, Meriwether C. Clotrimazole treatment of recurrent and chronic candida vulvovaginitis. Obstet Gynecol. 1989 Aug;73(3):330–4.

11. Crowley PD, Gallagher HC. Clotrimazole as a pharmaceutical: past, present and future. J Appl Microbiol. 2014 Sep;117(3):611–7.

12. Iavazzo C, Gkegkes ID, Zarkada IM, Falagas ME. Boric Acid for Recurrent Vulvovaginal Candidiasis: The Clinical Evidence. https://home.liebertpub.com/jwh. 2011 Aug;20(8):1245–55.

13. De Seta F, Schmidt M, Vu B, Essmann M, Larsen B. Antifungal mechanisms supporting boric acid therapy of Candida vaginitis. J Antimicrob Chemother. 2009;63(2):325–36.

14. Farr A, Effendy I, Frey Tirri B, Hof H, Mayser P, Petricevic L, et al. Guideline: Vulvovaginal candidosis (AWMF 015/072, level S2k). Mycoses. 2021;64(6):583–602.

15. Fan S, Liu X, Wu C, Xu L, Li J. Vaginal Nystatin Versus Oral Fluconazole for the Treatment for Recurrent Vulvovaginal Candidiasis. Mycopathologia. 2015 Feb;179(1–2):95–101.
