# Supplementary material for: Management of recurrent vulvovaginal candidosis: Narrative review of the literature and European expert panel opinion
Source: Front Cell Infect Microbiol. 2022 Sep 9;12:934353. doi: 10.3389/fcimb.2022.934353 (PMC9504472; doi:10.3389/fcimb.2022.934353)
Supplement: Supplementary Table 4 — Disease-free, relapse, failure, and patient adherence, persistence and satisfaction with published treatment regimens for recurrent vulvovaginal candidosis. [file Table_4.docx]

**sUPLEMENTARY table 4. disease-free, relapse, failure, and PATIENT adherence, persistence and satisfaction with published treatment regimens for recurrent vulvovaginal candidosis**

| Reference | Therapeutic agent | Regimen | | Women disease-free at 12 months, n (%) | Women relapsed at 12 months, n (%) | % failure (symptoms/infection persistence) at 12 months | Adherence/persistence/satisfaction on regimen |
| --- | --- | --- | --- | --- | --- | --- | --- |
| Sobel et al. 2004  (1) | **Fluconazole** | Induction therapy (open label, all patients received treatment) | 150 mg  every 72h x 3 doses | Fluconazole group: 54/126 (42.9%)  Placebo group: 30/137 (21.9%),  (*p*< 0.001) | Fluconazole group: 72/126 (57.1%)  Placebo group: 107/137 (78.1%),  (*p*< 0.001) | The median time to clinical recurrence in the fluconazole group was 10.2 months after randomization, as compared with 4.0 months in the placebo group (p<0.001)  The median time to mycologic relapse in the fluconazole group was 8.4 months after randomization, as compared with 1.9 months in the placebo group (P<0.001) | Not reported |
|  |  | Maintenance (Randomized) | 150 mg PO of fluconazole or placebo  once a week for six months |  |  |  |  |
| Donders et al. 2008 (2)  Individualized decreasing-dose maintenance fluconazole regimen for RVVC (ReCiDiF trial) | **Fluconazole** | Induction therapy | 3 x 200mg fluconazole for 1 week | 82/117 (70%) (inclusive induction phase)  86/117 (77%) (starting from maintenance phase) | 24/117 (21%) | 60% of the women being recolonized  with *Candida* after 1 year  A mean of 0.5% women experienced  a clinical relapse with *Candida* vaginitis per week | Most women adhered well to the individualized regimen with degressive prophylactic treatment  At 1 year, 72% of the women  were still on maintenance therapy |
|  |  | Maintenance therapy | 200 mg fluconazole  once/week x 8 weeks  200 mg fluconazole  once/2 weeks x 4 months  200 mg fluconazole  once/month x 6 months |  |  |  |  |
| Bolouri et al. 2008 (3) | **Fluconazole** | Induction therapy | 150 mg  every 72h x 3 doses | Positive culture:  Fluconazole group: 25/32 (78.1%)  Placebo group: 28/32 (87.5%) | Fluconazole group: 23/32 (71.9%)  Placebo group: 26/32 (81.3%) | Not reported | Not reported |
|  |  | Maintenance therapy | 150 mg of fluconazole or placebo  once a week for six months |  |  |  |  |
| witt et al 2009 (4) | **Itraconazole** | Induction therapy | 200 mg itraconazole | Free of culture-  detectable *Candida*:  Group 1: 18/23 (78%)  Group 2: 19/25 (76%)  Group 3: 9/23 (39%) | Women in the CH group had a recurrence of vaginal *Candida* infection significantly earlier compared with women in the itraconazole and itraconazole plus  lactobacilli groups (log-rank test; p=0.002) | Not reported | Women in the CH group were significantly less satisfied than patients in the itraconazole group and the itraconazole plus lactobacilli group (59.2, 68.2 and 71.7 respectively; p< 0.001) |
|  |  | Maintenance therapy | Group 1: One 200-mg tablet/ month  Group 2: One 200-mg tablet/ month + Lactobacilli  Group 3: classic homeopathy (CH) |  |  |  |  |
| Fong 1992  (5) | **Itraconazole** | Induction therapy | Itraconazole 200 mg orally daily (100 mg twice daily) for 5 days or Clotrimazole vaginal ovules 200 mg (Canesten  3 mg) daily for 5 days, | Itraconazole group: 44/21 (19%)  Clotrimazole group 6/17 (35.3%) | Itraconazole group: 10/21 (47.6%)  Clotrimazole group 11/17 (64%) | Not reported | Not reported |
|  |  | Maintenance therapy | Itraconazole 200 mg twice weekly for 6 months or  Clotrimazole 200 mg twice  weekly for 6 months. |  |  |  |  |
| Topical treatment | | | | | |  |  |
| Sobel et al. 1989 (6) | Clotrimazole | Induction therapy | 500 mg one vaginal suppository weekly for 2 weeks | Only data at 6 months is available:  Clinical cured  Clotrimazole: 47%  Placebo: 33%,  Mycological cured:  Clotrimazole: 20%  Placebo: 5% | Not reported | Not reported | Not reported |
|  |  | Maintenance therapy | 500 mg one vaginal suppository monthly for 6 months |  |  |  |  |
| Roth et al. 1990 (7) | Clotrimazole | Induction therapy | 500 mg, single dose vaginal tablet | Recolonization rate:  Clotrimazole group: 84.9%  Placebo group: 86.2% | Clotrimazole group: 84.9%  Placebo group: 86.2% | Not reported | Not reported |
|  |  | Maintenance therapy | One 500 mg vaginal tablet monthly for 6 months |  |  |  |  |
| Guaschino et al. 2001  (8) | Boric acid (compounding) | Induction therapy | Itraconazole: 200 mg x 3 days or  Boric acid 300 mg daily in vaginal ovules x 14 days | 56% of positive cultures for *Candida* | Not reported | 6 women (54%) in  each group reported symptoms | Not reported |
|  |  | Maintenance therapy | Itraconazole: 200 mg/month for 6 months  Boric acid:  600-mg vaginal ovule administered once daily during menstruation (5-day/6months) |  |  |  |  |
| Fan et al. 2015 (9) | Nystatin | Induction therapy | 20 mµ/day of nystatin vaginal suppositories for 14 days or  150 mg of fluconazole/ day x 14 days | Mycological cure:  Nystatin group: 78/96 (81.25%)  Fluconazole group: 60/73 (82.19%)  Mycological cure rates of RVVC caused by *C. glabrata* were 64.3% (27/42) compared to 12.5% (2/16) in the fluconazole group.  Nystatin therapy was successful in 55% (5/9) patients, with RVVC caused by fluconazole-resistant *Candida*, whereas in the fluconazole group, initial therapy failed in all patients (n=7). | Not reported | Not reported | Not reported |
|  |  | Maintenance therapy | 20 mµ/day of vaginal nystatin 7 days before and after menstruation for 6 months or weekly 150 mg dose of oral fluconazole capsule for 6 months |  |  |  |  |
| PO: oral; RRVC, recurrent vulvovaginal vaginosis | | | | | | | |

References

1. Sobel JD, Wiesenfeld HC, Martens M, Danna P, Hooton TM, Rompalo A, et al. Maintenance Fluconazole Therapy for Recurrent Vulvovaginal Candidiasis. N Engl J Med. 2004 Oct;351(9):876–83.

2. Donders G, Bellen G, Byttebier G, Verguts L, Hinoul P, Walckiers R, et al. Individualized decreasing-dose maintenance fluconazole regimen for recurrent vulvovaginal candidiasis (ReCiDiF trial). Am J Obstet Gynecol. 2008 Dec 1;199(6):613.e1-613.e9.

3. Bolouri F, Tabrizi NM, Tanha FD, Niroomand N, Azmoodeh A, Emami S, et al. Effectiveness of fluconazole for suppressive maintenance therapy in patients with RVVC: A randomized placebo-controlled study. Iran J Pharm Res. 2009;8(4):307–13.

4. Witt A, Kaufmann U, Bitschnau M, Tempfer C, Özbal A, Haytouglu E, et al. Monthly itraconazole versus classic homeopathy for the treatment of recurrent vulvovaginal candidiasis: A randomised trial. BJOG An Int J Obstet Gynaecol. 2009 Oct 1;116(11):1499–505.

5. Fong IW. The value of chronic suppressive therapy with itraconazole versus clotrimazole in women with recurrent vaginal candidiasis. Sex Transm Infect. 1992;68(6):374–7.

6. Sobel JD, Schmitt C, Meriwether C. Clotrimazole treatment of recurrent and chronic candida vulvovaginitis. Obstet Gynecol. 1989 Aug 1;73(3):330–4.

7. Roth AC, Milsom I, Forssman L, Wåhlén P. Intermittent prophylactic treatment of recurrent vaginal candidiasis by postmenstrual application of a 500 mg clotrimazole vaginal tablet. Sex Transm Infect. 1990 Oct 1;66(5):357–60.

8. Guaschino S, Seta F De, Sartore A, Ricci G, Santo D De, Piccoli M, et al. Efficacy of maintenance therapy with topical boric acid in comparison with oral itraconazole in the treatment of recurrent vulvovaginal candidiasis. Am J Obstet Gynecol. 2001 Mar 1;184(4):598–602.

9. Fan S, Liu X, Wu C, Xu L, Li J. Vaginal Nystatin Versus Oral Fluconazole for the Treatment for Recurrent Vulvovaginal Candidiasis. Mycopathologia. 2015 Feb 1;179(1–2):95–101.
