# Supplementary material for: Management of recurrent vulvovaginal candidosis: Narrative review of the literature and European expert panel opinion
Source: Front Cell Infect Microbiol. 2022 Sep 9;12:934353. doi: 10.3389/fcimb.2022.934353 (PMC9504472; doi:10.3389/fcimb.2022.934353)
Supplement: Supplementary Table 5 — Investigational drugs: summary of mechanism of action and preliminary results. [file Table_5.docx]

**supplemmentary Table 5. investigational drugs: Summary of mechanism of action and preliminary results**

| **Agent** | **Mechanism of action** | **Preclinical results** | **Study, treatment (dose and duration)** | **Efficacy results** | **Safety results** |
| --- | --- | --- | --- | --- | --- |
| Oteseconazole (1) (2) | Strongly binds to CYP51 of *Candida* species and produces a strong type II difference spectrum, with a target affinity similar to that of clotrimazole, fluconazole, itraconazole and voriconazole. It does not bind to human CYP51 | Broad activity against *C. albicans* and *C. glabrata* as well as activity against less-common strains. For most species, oteseconazole was on average more than 40-fold more potent than fluconazole | Phase 1, 2 and 3 studies | Culture-verified recurrence occurred in 4.1% of patients on oteseconazole at 48-weeks of maintenance (52.2% with placebo)  At 3-month and 6-month follow-up, all patients who received oteseconazole maintenance showed mycologic cure | Well-tolerated in all dosing groups  Most common TEAEs (>5% of patients): urinary tract infection, bacterial vaginosis, sinusitis, headache, upper respiratory tract infection and nausea, all mild or moderate |
| Ibrexafungerp (3) (4) | It non-competitively inhibits the β-(1,3) D-glucan synthase enzyme, compromising the fungal cell wall by making it highly permeable, thereby disrupting osmotic pressure, which can lead to cell lysis | Broad *in vitro* activity against wild-type, azole-resistant, and echinocandin resistant  *C. glabrata* species | Phase 3, multicenter, randomized, double-blind, placebo-controlled superiority study  Oral ibrexafungerp 300 mg twice a day for 1 day | Data only available for VVC caused by *C. albicans*: (ibrexafungerp vs placebo)   - Clinical cure (50.5% [95/188] vs 28.6% [28/98]; *P*=0.001) - Mycological eradication (49.5% [93/188] vs 19.4% [19/98]; *P*<0.001) - Overall success (36.0% [64/178] vs 12.6% [12/95]   In RVVC, results are expected by December 2021 | AEs were primarily gastrointestinal and mild in severity |
| Rezafungin (5) (6) | Binding to the heteromeric glycosyltransferase enzyme complex present in the fungal cell membrane and blocking β-(1,3)-d-glucan synthesis. This disruption in the β-(1,3)-d-glucan synthesis results in a leaky and highly permeable cell wall | Potent *in vitro* activity against *Candida* isolates (*C. albicans, C. glabrata, C. parapsilosis* and *C. tropicalis*), including azole-resistant strains | Phase 2, randomized, multicenter, open-label, sponsor blinded, study  Rezafungin 3% gel and rezafungin 6% ointment applied for 1 day | Data only available for VVC  In subjects with non-albicans infection**, persistence of positive cultures at day 28 was observed in 75% of subjects in rezafungin group** and 100% of the fluconazole group | Most common AEs in VVC: bacterial vaginosis, nasopharyngitis, trichomoniasis, upper respiratory infection, and gastroenteritis |
| Fortified azoles (7) (8) (9) | Variable depending on combination. Eventually, the synergic effect reduces or prevents the induction of resistance of fungal cells | *In vitro*, fortified azoles demonstrated to be active against *Candida* spp. resistant to fluconazole.  They also reduced the viability of biofilms of *Candida* spp. | No data available | No data available | No data available |
| AEs: adverse events, VVC: vulvovaginal candidosis, RVVC: recurrent vulvovaginal candidosis, TEAEs: treatment emergent AEs | | | | | |

References

1. Sobel JD, Nyirjesy P. Oteseconazole: an advance in treatment of recurrent vulvovaginal candidiasis. Future Microbiol [Internet]. 2021 Dec [cited 2021 Dec 27];16(18):1453–61. Available from: https://pubmed.ncbi.nlm.nih.gov/34783586/

2. Sobel J, Donders D, Degenhardt T, K P, Curelop S, Ghannoum M, et al. Efficacy and Safety of Oteseconazole in Recurrent Vulvovaginal Candidiasis. N Engl J Med. 2022;1(8):1–13.

3. Schwebke JR, Sobel R, Gersten JK, Sussman SA, Lederman SN, Jacobs MA, et al. Ibrexafungerp versus placebo for vulvovaginal candidiasis treatment: a phase 3, randomized, controlled superiority trial (VANISH 303). Clin Infect Dis. 2021 Sep;ciab750.

4. Gamal A, Chu S, McCormick TS, Borroto-Esoda K, Angulo D, Ghannoum MA, et al. Ibrexafungerp, a Novel Oral Triterpenoid Antifungal in Development: Overview of Antifungal Activity Against Candida glabrata. Front Cell Infect Microbiol. 2021 Mar 11;11:Article 642358.

5. Nyirjesy P, Alessio C, Jandourek A, Lee JD, Sandison T, Sobel JD. CD101 Topical Compared With Oral Fluconazole for Acute Vulvovaginal Candidiasis: A Randomized Controlled Trial. J Low Genit Tract Dis [Internet]. 2019 Jul 1 [cited 2021 Nov 22];23(3):226–9. Available from: https://pubmed.ncbi.nlm.nih.gov/30893271/

6. Boikov DA, Locke JB, James KD, Bartizal K, Sobel JD. In vitro activity of the novel echinocandin CD101 at pH 7 and 4 against Candida spp. isolates from patients with vulvovaginal candidiasis. J Antimicrob Chemother. 2017 May 1;72(5):1355–8.

7. Tits J, Berman J, Cammue BPA, Thevissen K. Combining Miconazole and Domiphen Bromide Results in Excess of Reactive Oxygen Species and Killing of Biofilm Cells. Front Cell Dev Biol. 2021 Jan 21;8:617214.

8. de Andrade Neto JB, da Silva CR, Barroso FD, do Amaral Valente Sá LG, de Sousa Campos R, S Aires do Nascimento FB, et al. Synergistic effects of ketamine and azole derivatives on Candida spp. resistance to fluconazole. Future Microbiol. 2020 Feb 1;15:177–88.

9. Dennis EK, Garneau-Tsodikova S. Synergistic combinations of azoles and antihistamines against Candida species in vitro. Med Mycol. 2019 Oct 1;57(7):874–84.
